# Supplementary material for: Antibody-Mediated Immobilization of Virions in Mucus
Source: Bull Math Biol. 2019 Aug 29;81(10):4069–99. doi: 10.1007/s11538-019-00653-6 (PMC6764938; doi:10.1007/s11538-019-00653-6)
Supplement: Supplementary file 1 — Supplementary material 1 (pdf 26064 KB) [file 11538_2019_653_MOESM1_ESM.pdf]

1 Typical virion trajectories

We display the typical trajectories of the four different types of classified particles: Freely Diffusing, Immobilized, Subdiffusive, and Outlier in Figure 1. The Freely Diffusing class made up 45.5246% of the paths (1689/3707), Immobilized class made up 53.0618% of the paths (1967/3707), Subdiffusive class made up 0.3507% of the paths (13/3707), and the outlier class made up 1.0251% of the paths (38/3707),

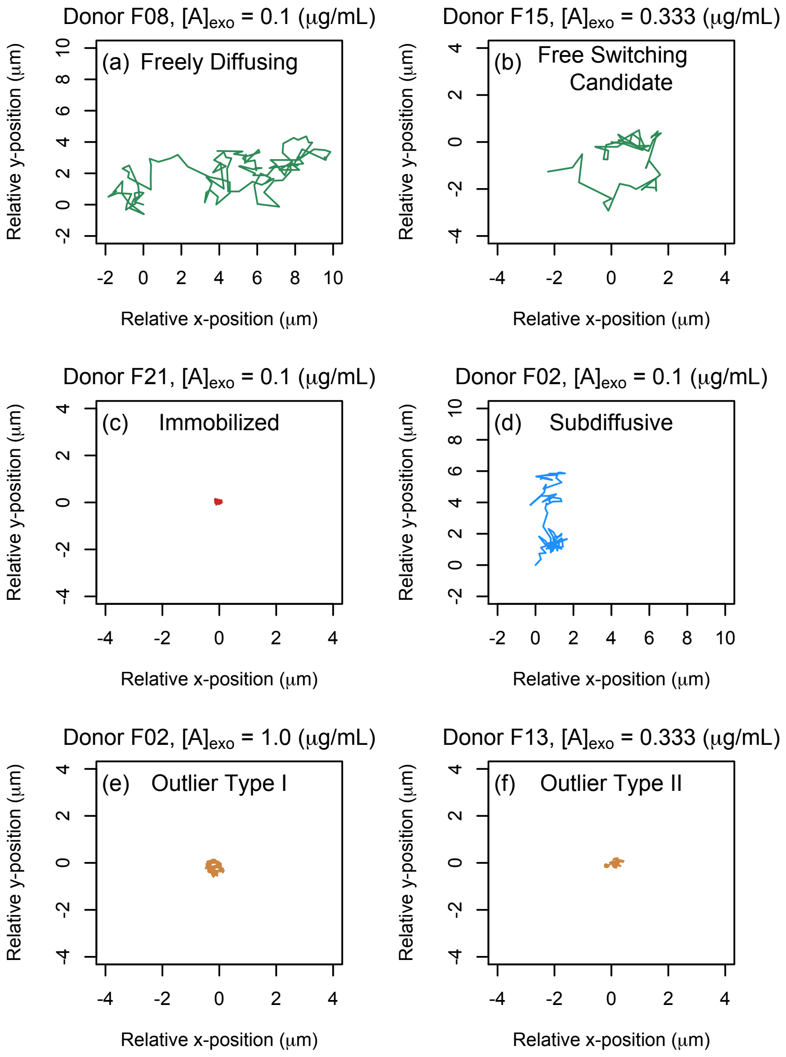

Fig. 1 **a-f**: A typical 2d-trajectory of a Virion classified as Freely Diffusing, Immobilized, Subdiffusive, Freely-Diffusing switch candidate, Outlier (Type I) and Outlier (Type 2), respectively.

2 Localization error and effective diffusivity

We used the method provided by Vestergaard et al. [1] (Section III.C) to estimate the variance of the localization error and the diffusivity of a freely diffusing particles for all tracked virions, under the assumption of maximal exposure time (motion blur coefficient = 1/6). In some cases where the virion has a correlated first-increment process (not freely diffusing), we obtained negative estimates for both the variance of the localization error and the diffusivity using the covariance based estimator. This was more common in the Immobilized class and the Freely Diffusing class had strictly positive diffusivity estimates using both methods.

The empirical distribution of the localization errors are provided in Figure 2(a) for the Immobilized and Freely Diffusing class in red and green, respectively. For those Freely Diffusing virions with positive localization error estimates, the estimate for the diffusivity using the covariance based estimator estimator was typical larger than the estimate for localization error by two orders of magnitude, as depicted in Figure 2(b). In Figure 2(c), we compare the estimate for the diffusivity using the maximum likelihood estimator (x-axis) and the estimator in [1], covariance based estimator, (y-axis).

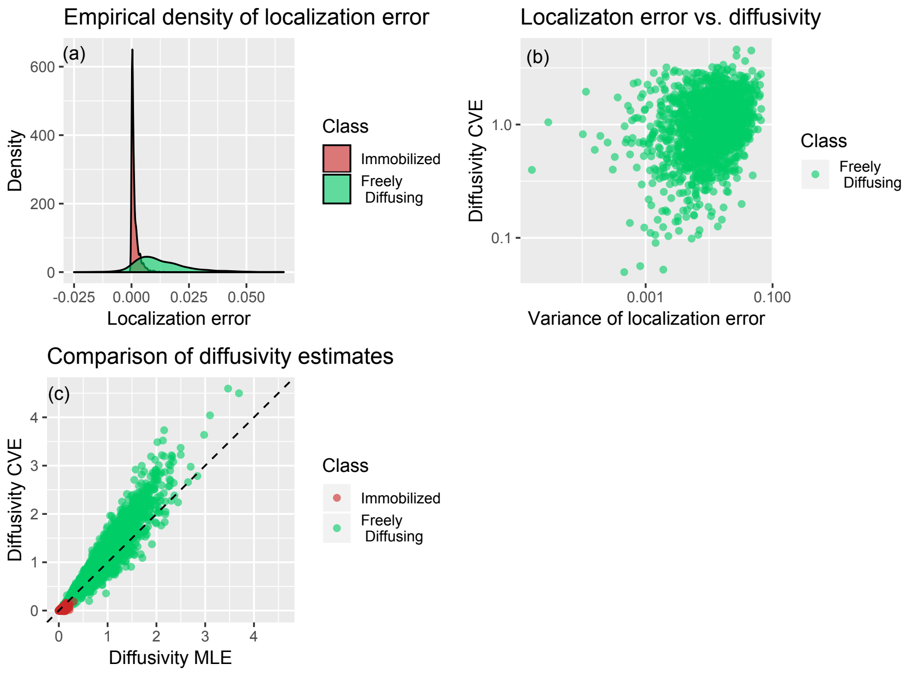

Fig. 2 **a**: The empirical density of the variance of the localization error for the Immobilized (red) and Freely Diffusing (green) sub-population. **b**: The diffusivity covariance based estimator (CVE) versus the estimate for the variance of the localization error for the Freely Diffusing class with positive estimates for the variance of the localization error on a log 10 vs. log 10 scale. **c**: The covariance based estimator (CVE) versus the maximum likelihood estimator (MLE) for the diffusivity for the Immobilized (red) and Freely Diffusing (green) class. The dashed black line denotes the line $x = y$.

3 Simulations of the immobilization process

We simulate the immobilization process, depicted in Figure 3 using a Gillespie algorithm. First, we simulate the number of number of bound Ab to the surface of a virion, $N(t)$. To do this, we use the transition rates given in Equation 8 to randomly select if a bound Ab is gained or lost and randomly sample the time the virion remains in the current state.

Then for the given value of $N(t) = n$, we simulate the number of simultaneously bound Ab, $S(t)$ for the duration of time $N(t)$ remains in given state. Because we assume the Ab-mucin dynamics are fast compared to Ab-virion dynamics, we approximate $S(t)$ by a continuous space process using the stochastic differential equation

$$d\tilde{S}(t) = \big(\lambda(\tilde{S}(t)) - \mu(\tilde{S}(t))\big)dt + \sqrt{(\lambda(\tilde{S}(t)) + \mu(\tilde{S}(t)))}dW(t) \tag{1}$$

where $\lambda(\tilde{S}(t)) = \big(N(t) - \tilde{S}(t)\big)g(\tilde{S}(t))m_{\text{on}}[M]$ (the rate of gaining a simultaneously bound Ab) and $\mu(\tilde{S}(t)) = \tilde{S}(t)m_{\text{off}}$ (the rate of losing a simultaneously bound Ab). Then we use the Euler–Maruyama method to simulate the process $\tilde{S}(t)$ for the duration of time the number of bound Ab remains fixed.

4 Clustering Algorithm Decision

For each donor we give a table of the classification of each cluster at each tested exogenous antibody concentration and graphically display the grouping of clusters based on the path-wise statistics. We provide a description of each of the four subpopulations in Table 1.

Table 1 Ensemble statistics for each of the four biological subpopulations.

	**Diffusing**	**Immobilized**		**Subdiffusive**	**Outlier**
$\langle D_{\text{eff}} \rangle$	$\geq 10^{-1}$	$< 10^{-1}$		$\geq 10^{-1}$	-
$\mathcal{A}(1;X), \mathcal{A}(1;Y)$	Independent	Independent	Anti-persistant	Anti-persistant	-
Motion	Brownian	Hindered Brownian	Stationary	Subdiffusive	-
Color	Green	Red		Blue	Brown

4.1 Donor F02

The dendrogram for Donor F02 was cut at a uniform height to yield four cluster for each tested exogenous Ab concentration except for $[A]_{\text{exo}} = 1.0\mu$g/mL, which was cut at a uniform height to yield five clusters because one particle was separated into its own cluster. The biological label that we assign to each cluster is reported in Table 2. We display the clusters based on the path-wise statistics in Figure 3, where the number corresponds to the hierarchical cluster and the color corresponds to the biological interpretation of the cluster.

Table 2 The classification of the clusters separated by the hierarchical clustering algorithm described in Section 2.3. Short hand notation: I = immobilized, F = Freely Diffusing, S = Subdiffusive, and O = Outlier. We indicate a member of a cluster was removed and classified as Outlier by $-/O$.

$[A]_{\text{exo}}(\mu\text{g/mL})$	Cluster 1	Cluster 2	Cluster 3	Cluster 4	Cluster 5
0	I	I	F	F	-
0.033	I	I	I	F	-
0.100	I	I	F/O	F	-
0.333	I	I	I	F	-
1.0	I	O	S/O	S	F

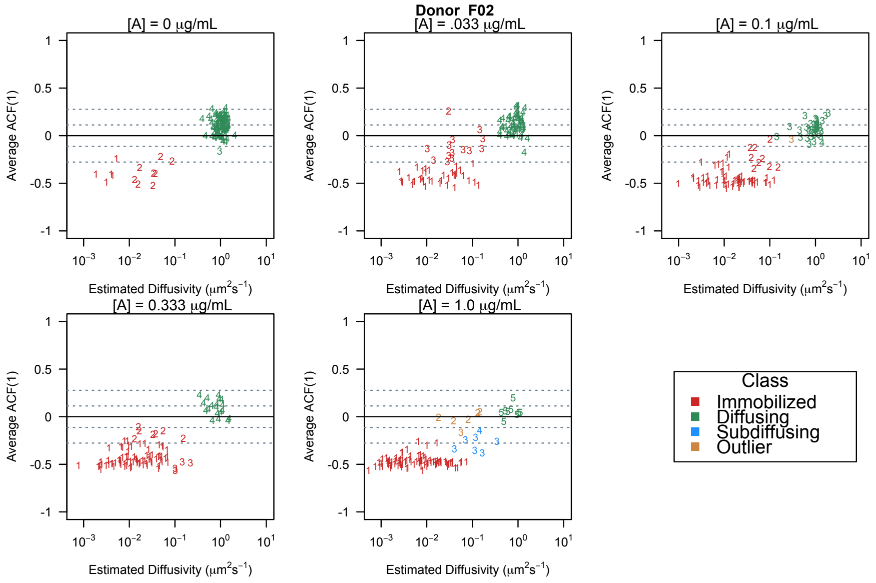

Fig. 3 **a-e** A depiction of the classification system, where each point, corresponds to a tracked virion with estimated diffusivity on a log 10 scale and average-ACF value, for Ab concentration 0, 0.033, 0.1, 0.333, and 1.0μg/mL, respectively. The numerical value of each point corresponds to the prescribed cluster by the hierarchical clustering algorithm and the color of the point represents the biological class.

4.2 Donor F05

The dendrogram for Donor F05 was cut at a uniform height to yield four cluster for each tested exogenous Ab concentration. The biological label that we assign to each cluster is reported in Table 3. We display the clusters based on the path-wise statistics in Figure 4, where the number corresponds to the hierarchical cluster and the color corresponds to the biological interpretation of the cluster.

Table 3 The classification of the clusters separated by the hierarchical clustering algorithm described in Section 2.3. Short hand notation: I = immobilized, F = Freely Diffusing, S = Subdiffusive, and O = Outlier. We indicate a member of a cluster was removed and classified as Outlier by $-/O$.

$[A]_{\text{exo}}$(μg/mL)	Cluster 1	Cluster 2	Cluster 3	Cluster 4
0	I	I	F	O
0.033	I	I	S	F
0.100	I	I	I	F
0.333	I	I	I	F
1.0	I	I	I	F

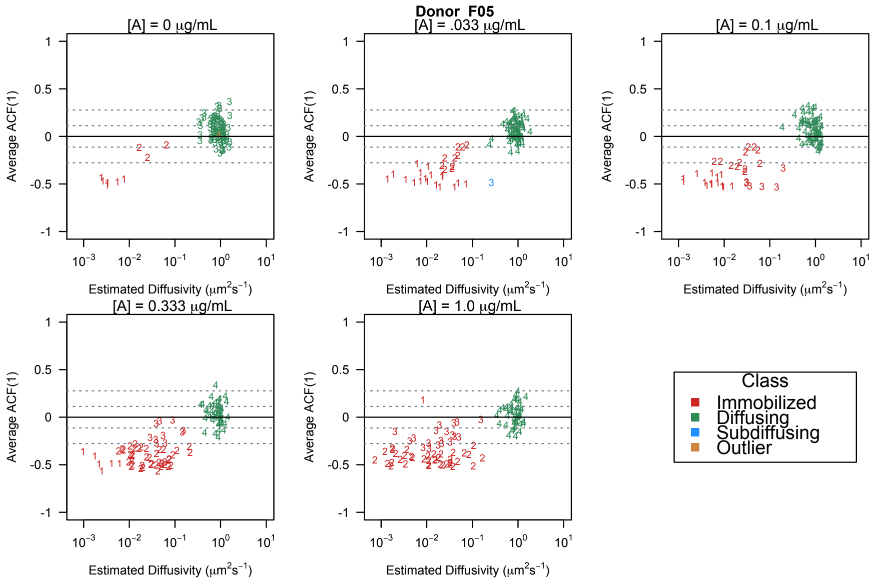

Fig. 4 **a-e** A depiction of the classification system, where each point, corresponds to a tracked virion with estimated diffusivity on a log 10 scale and average-ACF value, for Ab concentration 0, 0.033, 0.1, 0.333, and 1.0μg/mL, respectively. The numerical value of each point corresponds to the prescribed cluster by the hierarchical clustering algorithm and the color of the point represents the biological class.

4.3 Donor F08

The dendrogram for Donor F08 was cut at a uniform height to yield four cluster for each tested exogenous Ab concentration. The biological label that we assign to each cluster is reported in Table 4. We display the clusters based on the path-wise statistics in Figure 5, where the number corresponds to the hierarchical cluster and the color corresponds to the biological interpretation of the cluster.

Table 4 The classification of the clusters separated by the hierarchical clustering algorithm described in Section 2.3. Short hand notation: I = immobilized, F = Freely Diffusing, S = Subdiffusive, and O = Outlier. We indicate a member of a cluster was removed and classified as Outlier by $-/O$.

$[A]_{\text{exo}}(\mu\text{g/mL})$	Cluster 1	Cluster 2	Cluster 3	Cluster 4
0	I	F	F	F
0.033	I	I	F	F
0.100	I	I	S	F
0.333	I	I	F	F
1.0	I	I	S	F

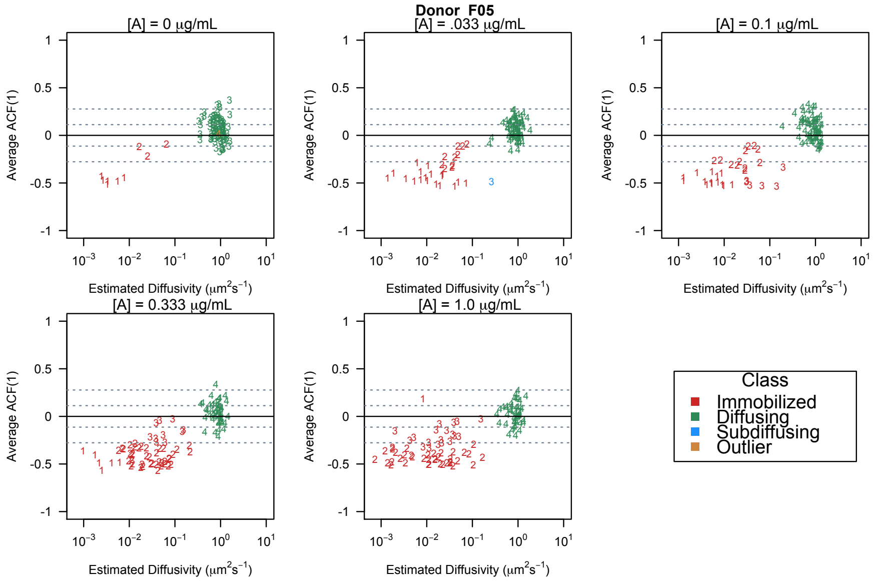

Fig. 5 **a-e**A depiction of the classification system, where each point, corresponds to a tracked virion with estimated diffusivity on a log 10 scale and average-ACF value, for Ab concentration 0, 0.033, 0.1, 0.333, and 1.0μg/mL, respectively. The numerical value of each point corresponds to the prescribed cluster by the hierarchical clustering algorithm and the color of the point represents the biological class.

4.4 Donor F13

The dendrogram for Donor F13 was cut at a uniform height to yield four cluster for each tested exogenous Ab concentration except for $[A]_{\text{exo}} = 1.0\mu\text{g/mL}$, which was cut at a uniform height to yield five clusters because one particle was separated into its own cluster. The biological label that we assign to each cluster is reported in Table 5. We display the clusters based on the path-wise statistics in Figure 6, where the number corresponds to the hierarchical cluster and the color corresponds to the biological interpretation of the cluster.

Table 5 The classification of the clusters separated by the hierarchical clustering algorithm described in Section 2.3. Short hand notation: I = immobilized, F = Freely Diffusing, S = Subdiffusive, and O = Outlier. We indicate a member of a cluster was removed and classified as Outlier by $-/O$.

$[A]_{\text{exo}}(\mu\text{g/mL})$	Cluster 1	Cluster 2	Cluster 3	Cluster 4	Cluster 5
0	I	I	S	F	F
0.033	I	I	F	F	F
0.100	I	I	O	S	F
0.333	I	I	I	F/O	F
1.0	I	I	I	F	F

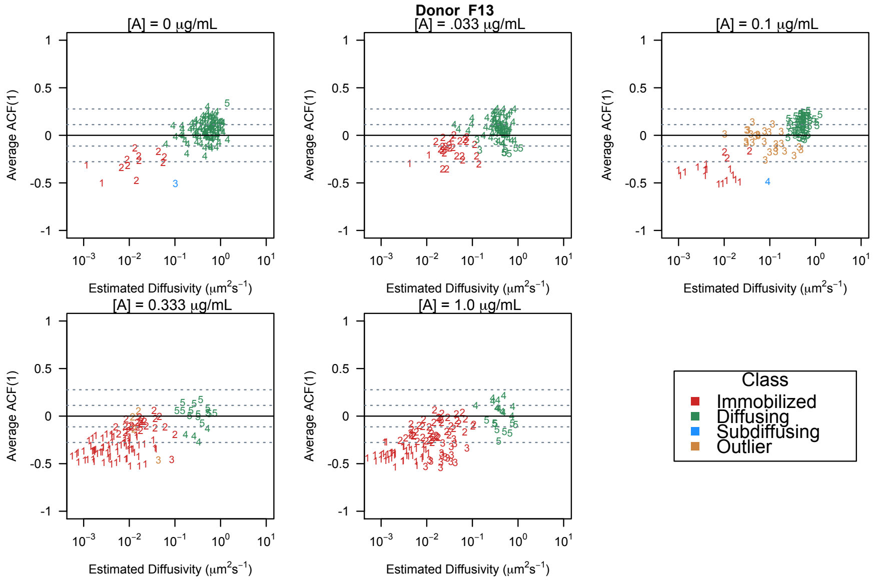

Fig. 6 **a-e** A depiction of the classification system, where each point, corresponds to a tracked virion with estimated diffusivity on a log 10 scale and average-ACF value, for Ab concentration 0, 0.033, 0.1, 0.333, and 1.0μg/mL, respectively. The numerical value of each point corresponds to the prescribed cluster by the hierarchical clustering algorithm and the color of the point represents the biological class.

4.5 Donor F15

The dendrogram for Donor F15 was cut at a uniform height to yield four cluster for each tested exogenous Ab concentration. The biological label that we assign to each cluster is reported in Table 6. We display the clusters based on the path-wise statistics in Figure 7, where the number corresponds to the hierarchical cluster and the color corresponds to the biological interpretation of the cluster.

Table 6 The classification of the clusters separated by the hierarchical clustering algorithm described in Section 2.3. Short hand notation: I = immobilized, F = Freely Diffusing, S = Subdiffusive, and O = Outlier. We indicate a member of a cluster was removed and classified as Outlier by $-/O$.

$[A]_{\text{exo}}(\mu$g/mL)	Cluster 1	Cluster 2	Cluster 3	Cluster 4
0	I	I	I	F
0.033	I	I	I	F
0.100	I	I	F	F
0.333	I	I	F	F
1.0	I	I	I	F

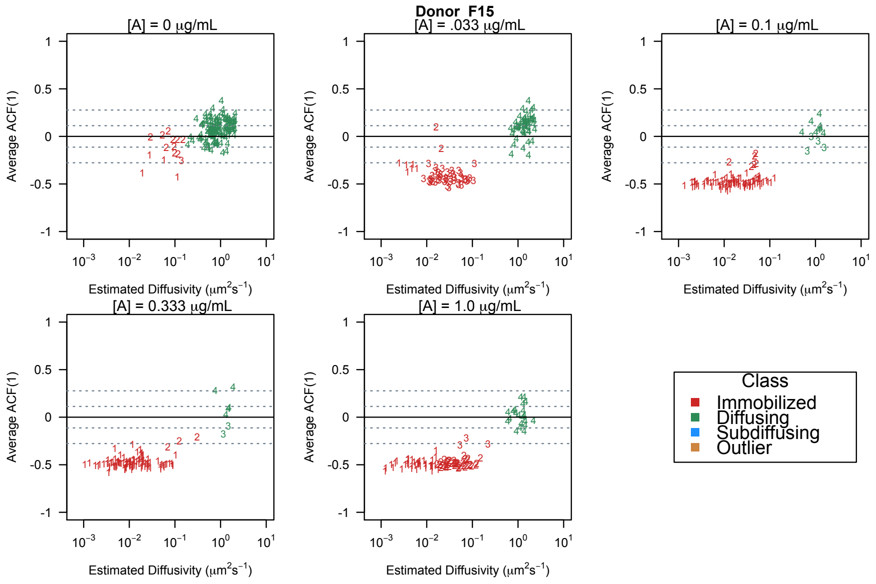

Fig. 7 **a-e** A depiction of the classification system, where each point, corresponds to a tracked virion with estimated diffusivity on a log 10 scale and average-ACF value, for Ab concentration 0, 0.033, 0.1, 0.333, and 1.0μg/mL, respectively. The numerical value of each point corresponds to the prescribed cluster by the hierarchical clustering algorithm and the color of the point represents the biological class.

4.6 Donor F17

The dendrogram for Donor F17 was cut at a uniform height to yield four cluster for each tested exogenous Ab concentration. The biological label that we assign to each cluster is reported in Table 7. We display the clusters based on the path-wise statistics in Figure 8, where the number corresponds to the hierarchical cluster and the color corresponds to the biological interpretation of the cluster.

Table 7 The classification of the clusters separated by the hierarchical clustering algorithm described in Section 2.3.. Short hand notation: I = immobilized, F = Freely Diffusing, S = Subdiffusive, and O = Outlier. We indicate a member of a cluster was removed and classified as Outlier by $-/O$.

$[A]_{\text{exo}}(\mu\text{g/mL})$	Cluster 1	Cluster 2	Cluster 3	Cluster 4
0	I	I	F	F
0.033	I	I	I	F
0.100	I	I	F	F
0.333	I	I	I	F
1.0	I	I	I	F

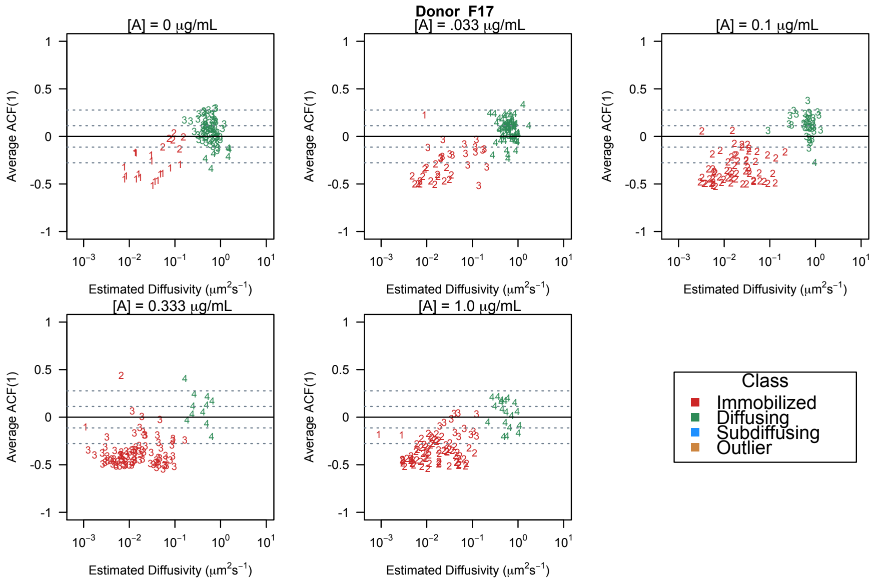

Fig. 8 **a-e** A depiction of the classification system, where each point, corresponds to a tracked virion with estimated diffusivity on a log 10 scale and average-ACF value, for Ab concentration 0, 0.033, 0.1, 0.333, and 1.0μg/mL, respectively. The numerical value of each point corresponds to the prescribed cluster by the hierarchical clustering algorithm and the color of the point represents the biological class.

4.7 Donor F21

The dendrogram for Donor 21 was cut at a uniform height to yield four cluster for each tested exogenous Ab concentration. The biological label that we assign to each cluster is reported in Table 8. We display the clusters based on the path-wise statistics in Figure 9, where the number corresponds to the hierarchical cluster and the color corresponds to the biological interpretation of the cluster.

Table 8 The classification of the clusters separated by the hierarchical clustering algorithm described in Section 2.3. Short hand notation: I = immobilized, F = Freely Diffusing, S = Subdiffusive, and O = Outlier. We indicate a member of a cluster was removed and classified as Outlier by $-/O$.

$[A]_{\text{exo}}(\mu$g/mL$)$	Cluster 1	Cluster 2	Cluster 3	Cluster 4
0	I	I	F	F
0.033	I	I	F	F
0.100	I	I	I	F
0.333	I	I	F	F
1.0	I	I	I	F

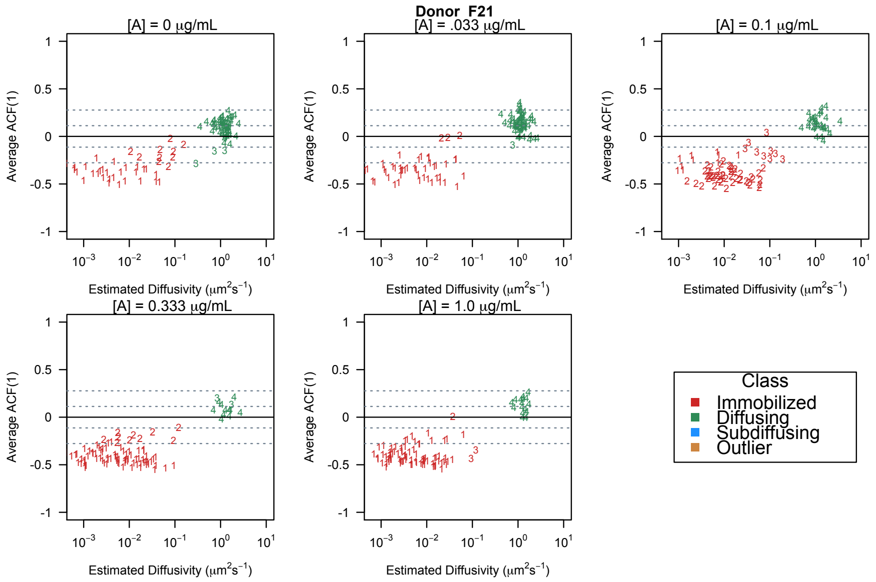

Fig. 9 **a-e** A depiction of the classification system, where each point, corresponds to a tracked virion with estimated diffusivity on a log 10 scale and average-ACF value, for Ab concentration 0, 0.033, 0.1, 0.333, and 1.0μg/mL, respectively. The numerical value of each point corresponds to the prescribed cluster by the hierarchical clustering algorithm and the color of the point represents the biological class.

5 Switch Point Detection Algorithm

5.1 Hamiltonian Monte Carlo (HMC) Sampling Algorithm

We used an HMC sampling algorithm to jointly estimate the parameters $(D, \tilde{\kappa}, \tau)$. Hamiltonian Monte Carlo is a form of Markov Chain Monte Carlo sampling that relies on Bayes Rule to sample from the joint posterior distribution of $(D, \tilde{\kappa}, \tau)$,

$$p(D, \tilde{\kappa}, \tau | \mathbf{x}, \mathbf{y}) \stackrel{c}{=} L(\mathbf{x}, \mathbf{y}; D, \tilde{\kappa}, \tau) p(D, \tilde{\kappa}, \tau) \tag{2}$$

where $L(\mathbf{x}, \mathbf{y}; (D, \tilde{\kappa}, \tau))$ is the likelihood of seeing the data given the parameters $(D, \tilde{\kappa}, \tau)$ and $p(D, \tilde{\kappa}, \tau)$ is the prior joint distribution of $(D, \tilde{\kappa}, \tau)$. In our model, we assume the parameters $D, \tilde{\kappa}$, and τ are independent so that the joint prior distribution factors into the product of priors, $p(D, \tilde{\kappa}, \tau) = p(D)p(\tilde{\kappa})p(\tau)$.

The likelihood function, $L(\mathbf{x}, \mathbf{y}; D, \tilde{\kappa}, \tau)$ is a consequence of the model. For the [diffusing → immobilized] switching scenario and [immobilized → diffusing] switching scenario $L(\mathbf{x}, \mathbf{y}; (D, \tilde{\kappa}, \tau))$ is given by Equation 16 and 15, respectively.

As for the priors, we placed a loosely informative prior on D

$$\pi(D) \sim \text{Gamma}(D_{\text{eff}} * 0.1, 0.1)$$

and chose a discrete uniform distribution over the observation times $\{t_n\}_{n=1}^{N-1}$ for the prior distribution on τ. Rather than directly sampling the parameter $\tilde{\kappa}$, we sampled the transform $\rho = \exp(-\Delta\tilde{\kappa})$ and then used the inverse transformation $\tilde{\kappa} = -\log(\rho)/\Delta$ to obtain posterior samples for $\tilde{\kappa}$. In doing this, we expressed our uncertainty of the order of $\tilde{\kappa}$ by placing a lognormal prior on ρ,

$$\pi(\rho) \sim \text{lognormal}\Big(loc = \log\big(\tfrac{0.5^2}{\sqrt{0.5^2+2}}\big), shape = \log(1 + \tfrac{2}{0.5^2})^{1/2}\Big).$$

We note that because τ is a discrete parameter taking values in $\{t_n\}_{n=1}^{N-1}$ whereas $D, \tilde{\kappa}$ are continuous real-valued parameters, posterior samples of $\tau | D, \tilde{\kappa}$ were first drawn from $\{t_n\}_{n=1}^{N-1}$ with probability

$$p(\tau = t_j | \mathbf{x}, \mathbf{y}, D, \tilde{\kappa}) = \frac{L(\mathbf{x}, \mathbf{y}; D, \tilde{\kappa}, \tau = t_j)p(\tau = t_j)}{\sum_{k=1}^{N} L(\mathbf{x}, \mathbf{y}; D, \tilde{\kappa}, \tau = t_k)p(\tau = t_k)} \quad \text{for } j = 1, \ldots N-1. \tag{3}$$

Then, we sampled D and $\tilde{\kappa}$ using the marginal posterior distribution of D and $\tilde{\kappa}$,

$$p(D, \tilde{\kappa} | \mathbf{x}, \mathbf{y}) \stackrel{c}{=} p(D)p(\tilde{\kappa}) \sum_{k=1}^{N} L(\mathbf{x}, \mathbf{y}; D, \tilde{\kappa}, \tau = t_k)p(\tau = t_k). \tag{4}$$

The HMC sampling algorithm was run using the `R` package rstan, and computations were done using high performance computing (HPC) resources and services provided by Technology Services at Tulane University, New Orleans, LA.

5.2 Testing Freely Diffusing population for switch candidates

Recall, we say a path is a candidate for switching if the 95% credible region for $\tau|D,\tilde{\kappa}$ is completely contained within the interval $[0.1\mathrm{T}_{\mathrm{final}}, 0.9\mathrm{T}_{\mathrm{final}}]$ where $\mathrm{T}_{\mathrm{final}}$ is the duration of a path. We tested if the i-th Freely Diffusing virion of path duration $\mathrm{T}_{\mathrm{final}}$, was a [immobilization → diffusion] switching candidate using the following procedure. Assuming the [immobilization → diffusion] switching model, we obtained posterior samples of $\tau|D,\tilde{\kappa}$ using our HMC sampling algorithm, in which we drew 2000 posterior samples and kept the last 1000 samples for 4 different runs of the algorithm. The resulting 4000 posterior samples of $\tau|_{D,\tilde{\kappa}}$ were used to construct the 95% credible interval for τ. If this interval was contained in $[0.1\mathrm{T}_{\mathrm{final}}, 0.9\mathrm{T}_{\mathrm{final}}]$, then we marked the virion as a candidate for [immobilization → diffusion] switching. We repeated this for all virions in the Freely Diffusing diffusing subpopulation to obtain the fraction of candidate for [immobilization → diffusion] switching. We obtained the fraction of candidate for [diffusion → immobilization] switching, following the same method described in the paragraph above but assuming the [diffusion → immobilization] switching model.

The Gaussian kernel density estimate of the posterior samples of τ within the 95% credible region for [immobilization → diffusion] switch candidates, and [diffusion → immobilization] switch candidates are displayed in top Figure 10a,b, respectively, for Donor F15. The path duration for each switch candidate is marked by a red x. In Figure 10c,d, we display the trajectory of an [immobilization → diffusion] switch candidates and [diffusion → immobilization] switch candidate, where the start of the trajectory by the green dot and the maximum a posteriori estimate for τ is marked by the blue star.

5.3 Estimating the false discovery rate

To estimate the false discovery rate of our switch detection test, we simulated Feely diffusing trajectories using parameters obtained from the class of Freely Diffusing virions in our data. To do this, the number of simulated Brownian paths was set to the number of virions classified as Freely diffusing, 1689 virions. Let $\mathbf{n}_{\mathrm{free}}$ and $\mathbf{D}_{\mathrm{free}}$ denote all the path lengths and the effective diffusivities of the Freely Diffusing virions, respectively. For the i-th simulated Brownian path, the path length n_i was sampled from $\mathbf{n}_{\mathrm{free}}$ and the diffusivity constant D_i was sampled from $\mathbf{D}_{\mathrm{free}}$.

Using our switch point criterion, we set the false discovery rate of [immobilization → diffusion] switches to the percent of simulated Brownian particles that were labeled as candidates for [immobilization → diffusion] switching for a likelihood function given by Equation 16. Specifically, the i-th simulated Brownian path and [immobilization → diffusion] switching model, the 95% credible region for τ was constructed using the 4000 posterior samples of $\tau|_{D,\tilde{\kappa}}$ obtained by the last 1000 (out of 2000) posterior samples of 4 runs of our HMC algorithm. We obtained an estimate for the false discovery rate of [diffusion → immobilization] switches in the same manner but assuming a [diffusion → immobilization] model.

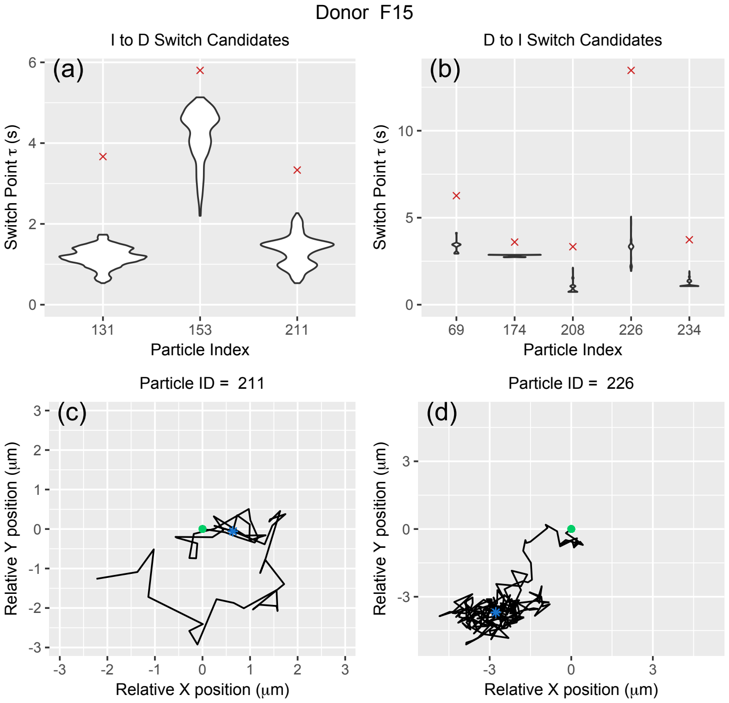

Fig. 10 **a,b** Violin plots for the posterior samples of τ within the 95% credible interval for those Freely-Diffusing virions from Donor F15 considered [immobilization $\rightarrow$ diffusion] switch candidates, and [Diffusion $\rightarrow$ Immobilization] switch candidate, respectively. **c** The 2d trajectory [immobilization $\rightarrow$ diffusion] switch candidate, (tracked particle 211 for Donor F15 at $[A]_{\text{exo}} = 0.333\mu$g/mL.) **d** The 2d trajectory [diffusion $\rightarrow$ immobilization] switch candidate, (tracked particle 226 for Donor F15 at $[A]_{\text{exo}} = 1.0\mu$g/mL.) In Panels (c) and (d) the green circle denotes the initial position of the virion and the blue star corresponds to the estimated switch point.

5.4 Estimating the power of the candidate switching test

In estimating the power of our test, we assumed that the switch point occurs within the middle 80% of the path. We believe this was a reasonable assumption because on average a Freely Diffusing virion had a path length of 80, and the first or last eight observations would be too noisy to capture the true dynamics of the virion. First, we created a simulated data set of 1689 [immobilization $\rightarrow$ diffusion] paths from the explicit solution of SDE given in Equation 15. For the ith [immobilization $\rightarrow$ diffusion] path, the model parameters $(n_i, D_i, \tilde{\kappa}_i, \tau_i)$ were sampled from the summary statistics of the tracked virions:

$$n_i \sim \text{Unif}\{\mathbf{n}_{\text{free}}\}, \quad D_i \sim \text{Unif}\{\mathbf{D}_{\text{free}}\}, \quad \tilde{\kappa}_i \sim \text{Exp}(\lambda = 1/500), \text{ and } \tau_i \sim \text{Unif}\{[0.1n_i, 0.9n_i]\}.$$

We ran our sampling algorithm assuming the model given by Equation 16 on the simulated [immobilization $\rightarrow$ diffusion] data. The sampling algorithm was run 4 times each for 2000 iterations where the last 1000 were kept as posterior samples.

We then set the power of our [immobilization → diffusion] switch point detecting test to the percentage of them have credible regions for τ within [$0.1T_{\text{final}}$, $0.9T_{\text{final}}$]. The test failed to detect switches in the following two cases: (1) The switch was located near the endpoints of the interval [$0.1T_{\text{final}}$, $0.9T_{\text{final}}$], resulting in a credible region outside the region allowed by the test. (2) When $\tilde{\kappa}$ is approximately less than 10. In this case, the immobilized state was indistinguishable from Brownian Motion because the deterministic component of the SDE given in Equation 16 was relatively small with respect to the random component. In Figure 11a,b, we display the relative position of the change point and $\tilde{\kappa}$ for simulated paths that were undetected by our proposed test. The trajectory of the simulated path marked by the pink diamond is shown in Figure 11c,d.

We estimated the power of [diffusion → immobilization] switch point detecting test following the same procedure as above but assumed the model given by Equation 15. For this switching scenario, switches went undetected due to the same reasons mentioned above.

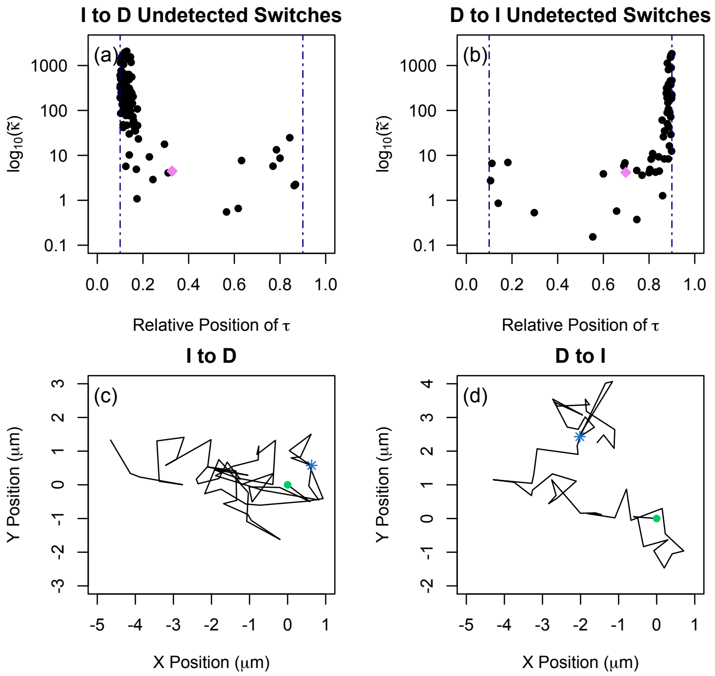

Fig. 11 **a,b** The true parameter $\tilde{\kappa}$ and the relative position of the switch point, τ, for the simulated switching particles, [immobilization → diffusion] and [diffusion → immobilized], respectively, whose switch point was undetected by our switching detection test. The dashed navy vertical lines mark the tenth percentile and ninetieth percentile of the trajectory. **c,d** The trajectory of a simulated switching particles marked by the pink diamond panels (a) and (b), respectively.

6 Statistical Evidence for trend in proportion immobilized

In Figure 12, we display the 95% BC_a confidence interval. for the observed proportion immobilized for each donor. We remark that for all donors, the proportion immobilized at the lowest tested exogenous Ab concentration (0μg/mL) and the highest tested exogenous Ab concentration (1μg/mL) have non-overlapping confidence intervals. In Table 9, we report the estimates coefficient and associated p-values for a negative exponential growth for the observed proportion of virions immobilized.

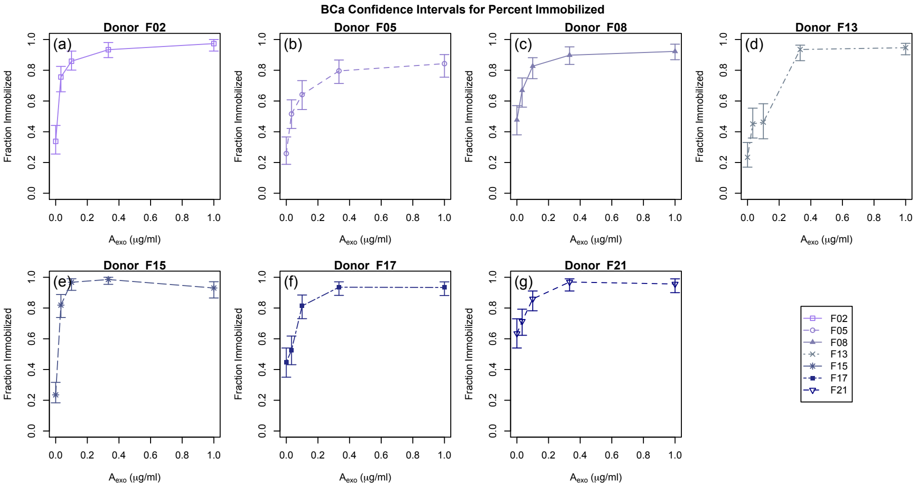

Fig. 12 **a-g** For each donor, the observed proportion immobilized with 95% BC_a confidence interval.

Table 9 Negative exponential growth coefficient estimates and associated p-values for the observed fraction of virions immobilized.

	Estimate	p -value
β_0	0.9138	< 0.0001
α_0	-0.8427	0.0043
α_{exo}	15.9201	< 0.0001

	Estimate	p -value
β_1	0.0642	0.4547
β_2	-0.1084	0.2123
β_4	-0.0700	0.4156
β_5	0.1140	0.1913
β_6	0.0050	0.9534
β_7	0.0347	0.6844

	Estimate	p -value
α_1	0.2846	0.3952
α_2	0.2272	0.5049
α_4	0.4286	0.1852
α_5	0.4385	0.1745
α_6	0.1901	0.5819
α_7	-0.2406	0.5763

7 Statistical Evidence for Effective Diffusivity

In Figure 13, we display the 95% BC_a confidence interval for the ensemble effective diffusivity for the Freely-Diffusing subpopulation. We remark that for all donors,

the ensemble effective diffusivity at the lowest tested exogenous Ab concentration (0μg/mL) and the highest tested exogenous Ab concentration (1μg/mL) have overlapping confidence intervals. We report the results of all the paired difference tests of the form,

$$H_0 : \langle D_{\text{eff}}([A]_i)\rangle - \langle D_{\text{eff}}([A]_j)\rangle = 0, \qquad H_A : \langle D_{\text{eff}}([A]_i)\rangle - \langle D_{\text{eff}}([A]_j)\rangle > 0. \quad (5)$$

for $1 \le i < j \le 5$ in Table 10.

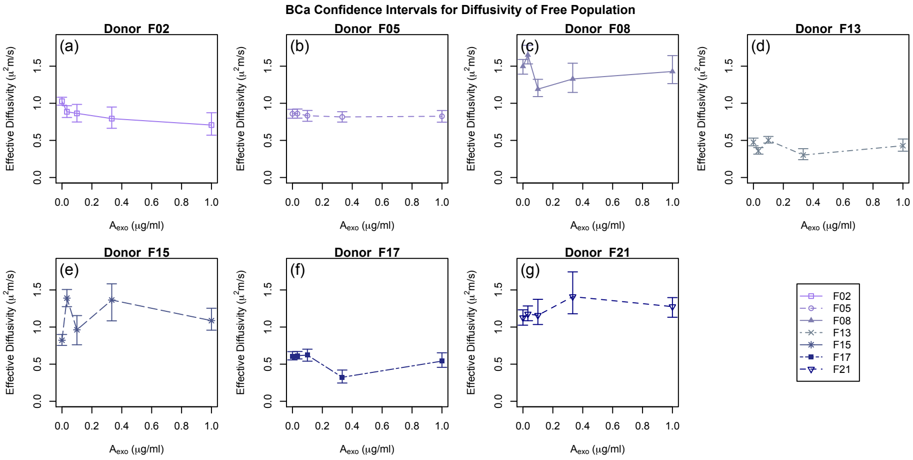

Fig. 13 a-g For each donor, the ensemble effective diffusivity of the free subpopulation with 95% BC_a confidence interval.

Table 10 The t-values of all possible combination of paired-difference tests of the form Equation 5. The critical value $t_{\alpha=0.05,6} = 1.943$.

	(1,2)	(1,3)	(1,4)	(1,5)	(2,3)	(2,4)	(2,5)	(3,4)	(3,5)	(4,5)
t-value	-0.7592	0.6669	0.0879	0.2325	1.2058	1.124	1.492	-0.2887	-0.3973	0.0931

8 Simple Linear Model

In 11, we report the range of each parameter for each donor and whether it was identifiable, practically unidentifiable, or structurally unidentifiable, under the assumption it takes one simultaneously bound Ab to immobilize a virion ($T = 1$) and the Ab-mucin binding rate is not affected by immobilization ($c = 1$).

Table 11 Parameter Identifiable assuming that $T = 1$ and $c = 1$ where ID indicates a structurally identifiable parameter and PU refers to a practically unidentifiable parameters. The confidence interval are given for each parameter where $\theta_{\alpha=0.95,3} = 7.814728$.

	$[A]_0$ (μg/mL)	N_*	q	$\min(\chi^2(\boldsymbol{\theta}))$
Donor F02	ID, $[0.006, 0.04]$	ID, $[140, 560]$	ID, $[0.89, 0.969]$	5.2106
Donor F05	ID, $[0.015, 0.085]$	ID, $[60, 260]$	ID $[0.76, 0.908]$	1.6292
Donor F08	ID, $[0.020, 0.115]$	ID, $[80, 360]$	ID $[0.86, 0.954]$	0.2755
Donor F13	ID, $[0.020, 0.075]$	ID, $[50, 100]$	PU $[0.93, 1)$	15.3627
Donor F15	ID, $[0.003, 0.01]$	ID, $[310, 780]$	ID $[0.93, .963]$	4.6863
Donor F17	ID, $[0.025, 0.095]$	ID, $[80, 210]$	ID $[0.90, .969]$	3.661
Donor F21	ID, $[0.035, 0.195]$	ID, $[70, 250]$	ID $[0.92, .989]$	1.277

9 Full Model

Let $\hat{\theta}$ denote the numeric estimate for $\boldsymbol{\theta} \in \Theta$ and $\hat{\theta}_{\text{slow}}$ denote the numeric estimate for $\boldsymbol{\theta}$ in the restricted parameter space $\Theta_{0.05,5} \cap \Theta_{\text{slow}}$. In 12, we report the range of each parameter for each donor within $\Theta_{0.05,5} \cap \Theta_{\text{slow}}$ and whether it was identifiable, practically unidentifiable, or structurally unidentifiable. We display the proportion immobilized predicted by our model assuming $\hat{\theta}_{\text{slow}}$ is the numeric estimate of the parameters restricted to the subspace $\Theta_{0.05,5} \cap \Theta_{\text{slow}}$ for each Donor in Figure 14.

Table 12 The values of T, c, and N_* that can permit $\boldsymbol{\theta} \in \Theta_{0.05,5} \cap \Theta_{\text{slow}}$. Lower and upper bounds of the tested parameter range are underlined. Note that $\chi^2(\alpha = 0.95, 5) = 11.0705$.

	T	c	N_*	$\chi^2(\hat{\theta})$)	$\chi^2(\hat{\theta}_{\text{slow}})$
Donor F02	$[19,\underline{60}$	$[44.67, \underline{10^4}]$	[120,620]	0.7070	9.1772
Donor F05	$[16,\underline{60}]$	$[79.43, \underline{10^4}]$	[90,370]	0.3912	4.1879
Donor F08	$[17,\underline{60}]$	$[56.23, \underline{10^4}]$	[100,540]	0.1562	1.2343
Donor F13	$\{13\}$	$[2818.38, \underline{10^4}]$	$\{70\}$	10.0055	18.0788
Donor F15	$[30,\underline{60}]$	$[44.68, \underline{10^4}]$	[250, 630]	4.2209	4.2209
Donor F17	[17,34	$[281.84, \underline{10^4}]$	[100, 200]	1.6432	3.0156
Donor F21	[19,27]	$[10^3, \underline{10^4}]$	[120,170]	0.5974	8.1838

References

1. Vestergaard, C.L., Blainey, P.C., Flyvbjerg, H.: Optimal estimation of diffusion coefficients from single-particle trajectories. Physical Review E **89**(2), 022726 (2014)

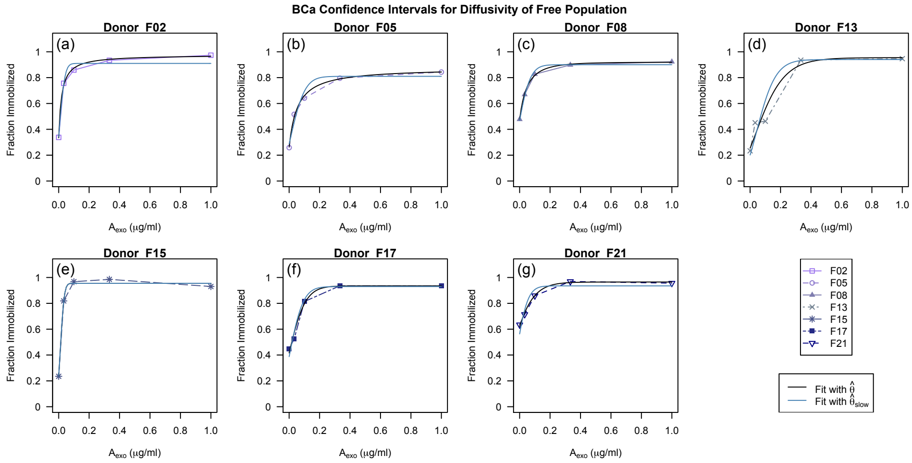

Fig. 14 a-g For each donor the observed proportion immobilized (dashed lines), proportion immobilized predicted by $\widetilde{\pi}([A];\hat{\theta})$, (solid black line), where $\hat{\theta}$ is the numeric estimate for $\boldsymbol{\theta}$ and the proportion immobilized predicted by $\widetilde{\pi}([A];\hat{\theta}_{\text{slow}})$ (solid blue line)), where $\hat{\theta}_{\text{slow}}$ is the numeric estimate restricted to the subspace $\Theta_{0.05,5} \cap \Theta_{\text{slow}}$. Note in Figure (e) $\hat{\theta}_{\text{slow}} = \hat{\theta}$.
